# A definition of free sugars for the UK

**DOI:** 10.1017/S136898001800085X

**Published:** 2018-06

**Authors:** Gillian E Swan, Natasha A Powell, Bethany L Knowles, Mark T Bush, Louis B Levy

**Affiliations:** Diet and Obesity Division, Health Improvement Directorate, Public Health England, Skipton House, 80 London Road, London SE1 6LH, UK

**Keywords:** Free sugars, Nutrient intake, Dietary recommendations National Diet and Nutrition Survey, UK Scientific Advisory Committee on Nutrition

## Abstract

Public Health England has set a definition for free sugars in the UK in order to estimate intakes of free sugars in the National Diet and Nutrition Survey. This follows the recommendation from the Scientific Advisory Committee on Nutrition in its 2015 report on *Carbohydrates and Health* that a definition of free sugars should be adopted. The definition of free sugars includes: all added sugars in any form; all sugars naturally present in fruit and vegetable juices, purées and pastes and similar products in which the structure has been broken down; all sugars in drinks (except for dairy-based drinks); and lactose and galactose added as ingredients. The sugars naturally present in milk and dairy products, fresh and most types of processed fruit and vegetables and in cereal grains, nuts and seeds are excluded from the definition.

In its 2015 report on *Carbohydrates and Health* ^(^ ^1^ ^)^, the UK Scientific Advisory Committee on Nutrition (SACN) recommended that a definition of ‘free sugars’ should be adopted in the UK for public health nutrition purposes, the concept replacing ‘non-milk extrinsic sugars’ on which sugar intake recommendations had been based for the last 25 years^(^ ^2^ ^)^. Free sugars as described by SACN comprise: ‘All monosaccharides and disaccharides added to foods by the manufacturer, cook or consumer, plus sugars naturally present in honey, syrups and unsweetened fruit juices. Under this definition, lactose (the sugar in milk) when naturally present in milk and milk products and the sugars contained within the cellular structure of foods are excluded’. This is very similar to the definition of free sugars published by the WHO in 2015^(^ ^3^ ^)^. SACN’s recommendation, that free sugars intake should not exceed 5 % of total dietary energy intake for adults and children from the age of 2 years, was adopted by the UK Government.

The National Diet and Nutrition Survey (NDNS) is the key data source used in the UK to monitor the diet and nutrition of the population in relation to recommendations and to provide the evidence base for development of policy at national level. To monitor progress towards the new free sugars recommendation, it is necessary to estimate intakes of free sugars in the NDNS. The nutrient databank includes about 6000 codes for foods and drinks, the vast majority being generic (covering a range of brands of a single product type). Each of these foods needs to be assigned a value for free sugars. In order to achieve this, and following further advice from SACN on processed fruit and vegetables^(^ ^4^ ^)^, Public Health England has expanded the broad definition set by SACN into a set of working principles for estimating the free sugars content of foods in a consistent, transparent way.

## Basis for Public Health England’s definition of free sugars

Public Health England’s definition of free sugars meets the need for a practical definition which can be readily applied to NDNS food codes using information available on product labels and which does not rely on detailed product specifications. It is based on:

1. the original definition set out by SACN in its report on *Carbohydrates and Health* ^(^ ^1^ ^)^;
2. further advice provided by SACN to Public Health England in 2016 on how the sugars naturally present in different types of processed fruit and vegetables should be classified with respect to free sugars^(^ ^4^ ^)^; and
3. the limited understanding of the extent to which the cellular structure of different types of processed foods containing naturally occurring sugars is broken down and the differences in the physiological response to sugar consumed in different forms.

Table 1 summarises the types and sources of sugars included in and excluded from the definition of free sugars.

**Table 1:** Free sugars definition: summary of inclusions and exclusions

Included in the definition of free sugars	Excluded from the definition of free sugars
All added sugar in whatever form, including honey, syrups and nectars whether added to products during manufacture or by the consumer during cooking or at the table. This includes ingredients such as malt extract and glucose syrup	Ingredients not included in the definition of sugar used for nutrition labelling, that is, monosaccharides and disaccharides Maltodextrins, oligofructose and sugar substitutes such as polyols (sorbitol) are excluded from the definition
Lactose and galactose added as an ingredient to foods or drinks, including lactose in whey powder added as an ingredient	Lactose and galactose when naturally present in milk and dairy products including milk powder
All the sugars naturally present in fruit and vegetable juices, concentrates, smoothies, purées, pastes, powders and extruded fruit and vegetable products	All the sugars naturally present in fresh and most types of processed (dried, stewed, canned and frozen) fruit and vegetables (including beans and pulses) except for juices, smoothies, purées, pastes, powders and extruded products Sugars naturally present in puréed and powdered potatoes and other starchy staples
All sugars in drinks except for milk and other dairy-based drinks. Including: ∙ all sugars in unsweetened fruit and vegetable juices, fruit and vegetable juice concentrates and smoothies; ∙ all sugars in alcoholic drinks; ∙ all sugars naturally present in dairy-alternative drinks such as soya, rice, oat and nut-based drinks	Lactose and galactose naturally present in milk and other dairy-based drinks
	All sugars naturally present in cereal grains including rice, pasta and flour regardless of processing (other than cereal-based drinks)
	All sugars naturally present in nuts and seeds regardless of processing (other than nut-based drinks)

Further details of the definition and the rationale for inclusions and exclusions are given below.

## Added sugars

Added sugars encompasses all monosaccharides and disaccharides added to foods. This includes: all types of cane and beet sugar, including both white and brown; sugar from other sources such as coconut palm sugar; crystalline sucrose, invert sugar, dextrose and molasses; fructose, sucrose, glucose, lactose, hydrolysed lactose and galactose added as an ingredient; the sugars in honey, treacle, malt extract and all types of syrups including glucose syrup, glucose–fructose syrup, high-fructose corn syrup and rice malt syrup; sugars in all types of nectars (examples are coconut blossom nectar; date nectar, agave nectar); and the sugars in unsweetened fruit or vegetable juices, juice concentrates, fruit or vegetable purées, pastes or jam added as an ingredient.

## Milk and other dairy products

In its report on *Carbohydrates and Health* ^(^ ^1^ ^)^, SACN stated that lactose as consumed in dairy products is excluded from the definition of free sugars as it has reduced cariogenicity compared with other sugars. Galactose in dairy products has also been excluded from the definition as specified by the WHO 2015 report on sugars^(^ ^3^ ^)^. Under this principle, lactose and galactose naturally present in dairy products used as an ingredient in composite foods containing liquid milk or milk powder, such as milk chocolate and ice cream, has not been included as free sugars. However, either lactose or galactose added to foods as an ingredient has been included in the definition of free sugars. This includes the lactose content of whey powder added as an ingredient.

## Processed fruit and vegetables

SACN advised that the sugars naturally present in fruit and vegetables that have been blended, pulped, puréed, extruded or powdered should be treated as free sugars on the basis that the cellular structure has been broken down^(^ ^4^ ^)^; but that the sugars naturally present in other types of processed fruit and vegetables (dried, canned (excluding juice or syrup), stewed, pressed) fall outside the definition of free sugars. Fruit and vegetables in some products are processed by more than one method. If one of the methods used is included in the definition of free sugars (Table 1), then the sugar in the fruit or vegetable is treated as free sugars. So, for example, dried fruit which has been puréed or extruded would be included as free sugars. SACN further advised that there was no scientific basis for treating the sugars in vegetables differently from the sugars in fruit. On this basis, the definition of free sugars includes the sugars in tomato purée and other vegetable purées, pastes and powders, fruit purées, pastes and powders and the sugars in extruded fruit products, but not products made with pressed dried fruit.

The definition of fruit and vegetables follows that used for the Eatwell Guide^(^ ^5^ ^)^ and its 5 A Day public health messaging, so sugars in powdered or puréed potatoes were considered to fall outside the definition, that is, the sugars present are not free sugars. The sugars in other starchy staples, such as powdered or puréed sweet potatoes, were also treated as outside the definition. The sugars in puréed or powdered beans or pulses, such as hummus, were included in the definition of free sugars as beans and pulses were included in 5 A Day.

## Drinks

All sugars in drinks, with the exception of lactose and galactose naturally present in milk and other dairy-based drinks, are defined as free sugars. This includes the sugars in soft drinks, fruit and vegetable juices and smoothies, alcoholic drinks and also the sugars naturally present in dairy-alternative drinks such as soya, nut, rice and oat drinks. The basis for including all the sugars in drinks in the definition is that drinks have the potential to deliver large amounts of sugar and they have lower satiety effects than do solid foods^(^ ^6^ ^)^.

## Assumptions used to estimate free sugars in the National Diet and Nutrition Survey

The free sugars content of NDNS food codes have been estimated from recipes based on ingredient information from product labels, using the definition described above. To keep the process manageable, assumptions were made about the extent to which the cellular structure of fruit and vegetables present in composite processed products had been broken down. For example, all sugars in jams, conserves, fruit spreads and preserves were treated as free sugars on the basis that the cellular structure of the fruit in such products is predominantly broken down and the proportion of sugars naturally present from the fruit is small in comparison to the amount of added sugar. For soups containing vegetables it was assumed that all the vegetables were puréed or blended (as in a smooth soup) unless it was feasible to estimate the proportion of intact vegetables based on knowledge of the range of products available. The same approach was used for fruit yoghurts and similar products.

These are examples of some of the assumptions used to assign free sugars values to NDNS food codes. A manufacturer estimating the free sugars content of a product would have access to more detailed, quantitative information on the ingredients used and the extent to which they have been processed. In these circumstances some of the assumptions outlined above may not be necessary. Using a more detailed, quantitative ingredient specification will give a more accurate assessment of free sugars content.

Estimates of free sugars intakes based on the above definition are due to be published in the next NDNS report (Years 7 & 8) in 2018. This report will include a more detailed description of the approach used to estimate the free sugars content of food codes in the NDNS, based on the definition set out here.
